# Higher body mass index indicated better overall survival in pancreatic ductal adenocarcinoma patients: a real-world study of 2010 patients

**DOI:** 10.1186/s12885-021-09056-0

**Published:** 2021-12-09

**Authors:** Ningzhen Fu, Yu Jiang, Kai Qin, Hao Chen, Xiaxing Deng, Baiyong Shen

**Affiliations:** 1 Department of General Surgery, Pancreatic Disease Center, Shanghai Ruijin Hospital affiliated with Shanghai Jiao Tong University School of Medicine, No.197 Ruijin Er Road, Shanghai, 200025 China; 2 Research Institute of Pancreatic Diseases, Shanghai Jiao Tong University School of Medicine, No.197 Ruijin Er Road, Shanghai, 200025 China; 3 State Key Laboratory of Oncogenes and Related Genes, No.197 Ruijin Er Road, Shanghai, 200025 China; 4 Institute of Translational Medicine, Shanghai Jiao Tong University, Shanghai, China

**Keywords:** Body mass index (BMI). Pancreatic ductal adenocarcinoma (PDAC). Overall survival (OS). Restricted cubic spline (RCS). Chemotherapy

## Abstract

**Background:**

The association between body mass index (BMI) and the overall survival (OS) of pancreatic ductal adenocarcinoma (PDAC) patients remains controversial and unclear,

**Method:**

A total of 2010 patients from a high-volume center were enrolled in the study. The OS of PDAC patients was evaluated based on restricted cubic spline (RCS), propensity score (PS) and multivariable risk adjustment analyses.

**Result:**

BMI was linearly related to the OS (total *P* = 0.004, nonlinear *P* = 0.124). BMI was analyzed as categorical data based on X-tile software-defined cutoffs and World Health Organization (WHO)-recommended cutoffs. Adjusted with confounding covariates, higher BMI manifested as a positive prognostic predictor. Furthermore, BMI was proven to be associated with the OS in the PS analysis. (Underweight_Xtile_ vs. Normal_Xtile_*P* = 0.003, Overweight_Xtile_ vs. Normal_Xtile_*P* = 0.019; Underweight_WHO_ vs. Normal_WHO_*P* < 0.001, Overweight_WHO_ vs. Normal_WHO_*P* = 0.024). It was also revealed that patients with higher BMI benefitted more from chemotherapy. (Adjusted hazard ratio (aHR): Underweight_Xtile_ vs. Normal_Xtile_ vs. Overweight_Xtile_: 0.565 vs. 0.474 vs. 0.409; Underweight_WHO_ vs. Normal_WHO_ vs. Overweight_WHO_: 0.613 vs. 0.464 vs. 0.425).

**Conclusion:**

Among PDAC patients, there was a positive association between BMI and the OS, especially in patients treated with chemotherapy.

**Supplementary Information:**

The online version contains supplementary material available at 10.1186/s12885-021-09056-0.

## Background

Pancreatic cancer, one of the most lethal malignancies, is estimated to be responsible for 47,050 deaths in 2020 [1]. The 5-year survival rate is only 9% [1]. Pancreatic ductal adenocarcinoma (PDAC) is the main pattern of pancreatic cancer, accounting for approximately 85% [2]. How to predict and improve the overall survival (OS) of these patients is an long-standing focus of clinicians and researchers.

Body mass index (BMI), an easily accessible and inexpensive parameter, has been reported to be associated with the incidence of pancreatic cancer [3, 4]. In our previous study, it was discovered that there was an association between higher BMI and better OS among PDAC patients [5]. Thus far, the role of BMI in the long-term prognoses of PDAC patients remains controversial [6–10]. Limited by sample size and debatable statistics, in what way BMI affects the OS was not convincingly determined in previous studies. In this study, relying on propensity score (PS) analysis and multivariable risk adjustment analysis, we sought for the association between BMI and the OS. Based on our results, further studies could be designed to explore the underlying mechanisms and relationships between metabolic status and PDAC.

## Methods

### Data collection

All eligible patients pathologically diagnosed with PDAC were consecutively enrolled from the Pancreatic Disease Center at the Ruijin Hospital Affiliated to Shanghai Jiao Tong University School of Medicine from 2013.1 to 2019.12. The exclusion criteria were as follows: 1) no BMI data at diagnosis, 2) incomplete oncological data, 3) no regular follow-up, and 4) heterogenous carcinoma. Finally, 2010 patients were included in our study. (Supp. Figure 1).

The study protocol was approved by the institutional review board at the authors’ affiliated hospital. The local ethics committee waived the need for informed consent because the study was observational and retrospective. The study was undertaken according to the Strengthening the Reporting of Observational Studies in Epidemiology (STROBE) guidelines [11] and in accordance with the latest version of the Declaration of Helsinki.

### Definition

Height and weight were recorded at diagnosis. Body mass index was calculated as weight in kilograms divided by the square of height in meters. The cutoffs defined by X-tile were 18.9 kg/m^2^ and 23.3 kg/m^2^ for trinomial categorization. Additionally, BMI was categorized into three groups based on the cutoffs (18.5 kg/m^2^ and 23 kg/m^2^) defined by the World Health Organization (WHO) for Asians [12]. Patients under 18.9 kg/m^2^, between 18.9–23.3 kg/m^2^ and over 23.3 kg/m^2^ were respectively marked as underweight, normal and overweight. Similar categorization was performed based on WHO cutoffs. For better discrimination, different categorizing standards were marked using subscript text (−_Xtile_ or -_WHO_). Disease was staged per the 8th American Joint Committee on Cancer (AJCC) guidelines using the TNM system. Treatment strategies were determined per NCCN guidelines. Surgeries were performed for patients with resectable tumors. For patients with locally advanced and borderline-resectable disease, treatment with neoadjuvant chemotherapy with or without subsequent surgical resection or upfront surgery was provided. For patients with metastatic disease, palliative and supportive care was provided. Biliary drainage included preoperative biliary stent placement, nasobilliary drainage and percutaneous transhepatic cholangial drainage. The chemotherapy mentioned in the *RESULTS* and *DISCUSSION* sections refers to the postoperative adjuvant chemotherapy strategy or the chemotherapy strategy for patients with unresectable or metastatic disease. The regimens of the postoperative chemotherapy strategy included gemcitabine only, gemcitabine+ S-1, gemcitabine+ capecitabine, gemcitabine+ oxaliplatin and gemcitabine+ nimotuzumab. The regimens used for patients with advanced disease were albumin paclitaxel+ gemcitabine and FOLFIRINOX. Overall survival (OS) was calculated in months and defined as the time from diagnosis to the date of death due to any cause.

### Statistical analysis

Normally distributed continuous variables are presented as the mean ± SD and were analyzed using Student’s t-test. Nonnormally distributed continuous variables are presented as medians (quartile 1-quartile 3 (Q1-Q3)) and were analyzed using the Mann-Whitney U test. The Kolmogorov-Smirnov test was utilized for normality tests of continuous variables. Categorical variables are presented as percentages and were analyzed using Pearson’s test. The proportional hazards assumption (PH assumption) was tested using the Schoenfeld partial residual method [13]. The OS was assessed using Kaplan-Meier curves and log-rank tests in the univariable analyses and restricted cubic spline (RCS) curves (continuous BMI variable) [14–16] and Cox proportional hazards models (continuous and categorical BMI variables) were used for multivariable analyses adjusted with the clinically relevant factors identified in the univariable analyses. The Cox-Mantel test was used for significance comparison. For better result presentation, in Cox regression models, CA199 was transformed into log (CA199). Moreover, due to the coherence of surgical intervention and TNM stages, surgical intervention was hidden in the multivariable Cox models and PS analyses. The knot number for RCS curves was set as 5 because of the sample size. X-tile was utilized to determine the cutoff value [17]. *Associations* (statistical “association” was marked as italic text) between categorical data were assessed using the χ2 test, *associations* between continuous data were assessed using the Spearman rank test, and *associations* between categorical data and continuous data were assessed using the Kruskal-Wallis test. For *interaction* (statistical “interaction” was marked as italic text) evaluation, the product term in the Cox model was utilized to assess the multiplicative *interaction*, while the relative excess risk due to interaction (RERI), the attributable proportion due to interaction (AP) and the synergy index (S) were used for the assessment of additive *interaction* [18, 19]. If there is no biological interaction, RERI and AP are equal to 0 and S is equal to 1.

To eliminate the imbalance of chemotherapy administration among BMI categories, inverse probability of treatment weighting (IPTW) and standardized mortality ratio weighting (SMRW) were also introduced for further analysis. The matching parameters included age, sex, ASA physical status, CA199, total bilirubin (TB), fasting blood glucose (FBG), albumin (ALB), biliary drainage, stage, differentiation and chemotherapy administration.

Statistical analyses were performed by SPSS (IBM SPSS Statistics 26.0), R Studio and X-Tile software. *P* < 0.050 was regarded to be statistically significant.

## Results

### Patients and characteristics

2010 patients were enrolled in this study, and the baseline data were displayed in Table 1 and Supp. Table 1. The median BMI was 22.67 kg/m^2^. The median OS time was 17.3 months, while the median follow-up duration was 33.3 months. Chemotherapy administered to 56.5% of the patients. The whole cohort consisted of 192 Ia (9.6%), 478 Ib (23.8%), 155 IIa (7.7%), 532 IIb (26.5%), 432 III (21.5%) and 221 IV (11%) stage patients. For the X-tile categorization, the underweight, normal and overweight groups consisted of 216 (10.7%), 968 (48.2%) and 826 (41.1%) patients, respectively. For the WHO categorization, these three categories included 157 (7.8%), 943 (46.9%) and 910 (45.3%) patients, respectively. Significant differences were observed in age, sex, FBG and chemotherapy administration among the categories when analyzed with both strategies.

**Table 1:** Demographic and baseline characteristics of study cohort. (categorized by X-tile cutoffs)

	Underweight (*n* = 216)	Normal (*n* = 968)	Overweight (*n* = 826)	total	*P* value
Age	65 (59–72)	63 (57–69)	64 (57–69)	63 (58–69)	0.004
Male (%)	117 (54.2)	583 (60.3)	551 (66.7)	1251 (62.2)	0.001
ALB	38 (35–42)	39 (36–42)	40 (36–43)	39 (36–42)	0.001
FBG	5.68 (5.00–6.79)	6.04 (5.35–7.41)	6.10 (5.43–7.55)	6.03 (5.32–7.40)	< 0.001
CA199	165.3 (37.1–766.9)	152.2 (39.0–484.6)	163.8 (41.7–563.7)	161.1 (40.2–552.2)	0.34
TB	16.9 (11.4–75.8)	17.0 (11.3–76.7)	16.6 (11.8–70.3)	16.8 (11.6–72.6)	0.935
Biliary drainage (%)	38 (24.5)	152 (20.8)	134 (21.8)	324 (21.6)	0.486
ASA Score (%)					0.394
1	130 (61.6)	530 (56.5)	433 (53.9)	1093 (56)	
2	65 (30.8)	334 (35.6)	297 (37)	696 (35.7)	
3	14 (6.6)	63 (6.7)	67 (8.4)	144 (7.4)	
4	2 (0.9)	11 (1.2)	6 (0.7)	19 (1)	
Tumor location (%)					0.495
Head	129 (59.7)	538 (55.6)	473 (57.2)	1140 (56.7)	
Body/Tail	87 (40.3)	430 (44.4)	353 (42.8)	870 (43.3)	
Diagnostic year (%)					0.758
2013	9 (4.2)	35 (3.6)	32 (3.9)	76 (3.8)	
2014	19 (8.8)	57 (5.9)	56 (6.8)	132 (6.6)	
2015	22 (10.2)	105 (10.8)	80 (9.7)	207 (10.3)	
2016	30 (13.4)	146 (15.1)	137 (16.6)	313 (15.6)	
2017	48 (22.7)	199 (20.6)	149 (18.0)	396 (19.7)	
2018	47 (21.8)	205 (21.3)	174 (21.1)	426 (21.2)	
2019	41 (19)	221 (22.8)	198 (24.0)	460 (22.9)	
Stage					0.162
Ia	17 (8.9)	88 (9.2)	87 (10.4)	192 (9.6)	
Ib	48 (22.2)	222 (22.9)	208 (25.2)	478 (23.8)	
IIa	19 (8.8)	72 (7.4)	64 (7.8)	155 (7.7)	
IIb	59 (27.3)	248 (25.6)	225 (27.3)	532 (26.5)	
III	48 (22.2)	238 (24.6)	146 (17.7)	432 (21.5)	
IV	25 (11.3)	100 (10.3)	96 (11.6)	221 (11)	
Differentiation (%)					0.252
I	0 (0)	6 (0.6)	1 (0.1)	7 (0.3)	
II	67 (31)	325 (33.6)	258 (31.2)	650 (32.3)	
III	149 (69)	633 (65.3)	567 (68.7)	1349 (67.1)	
IV	0 (0)	1 (0.1)	0 (0)	1 (0)	
Unknown	0 (0)	3 (0.3)	0 (0)	3 (0.1)	
Chemotherapy (%)	93 (43.1)	57 (56.9)	500 (60.5)	1144 (56.9)	< 0.001
Surgical interventions (%)	152 (70.4)	669 (69.1)	598 (72.4)	1419 (70.6)	0.313

*ALB* Albumin, *FBG* Fasten blood glucose, *TB* Total bilirubin

### Relationship of BMI with OS

**Fig. 1:**
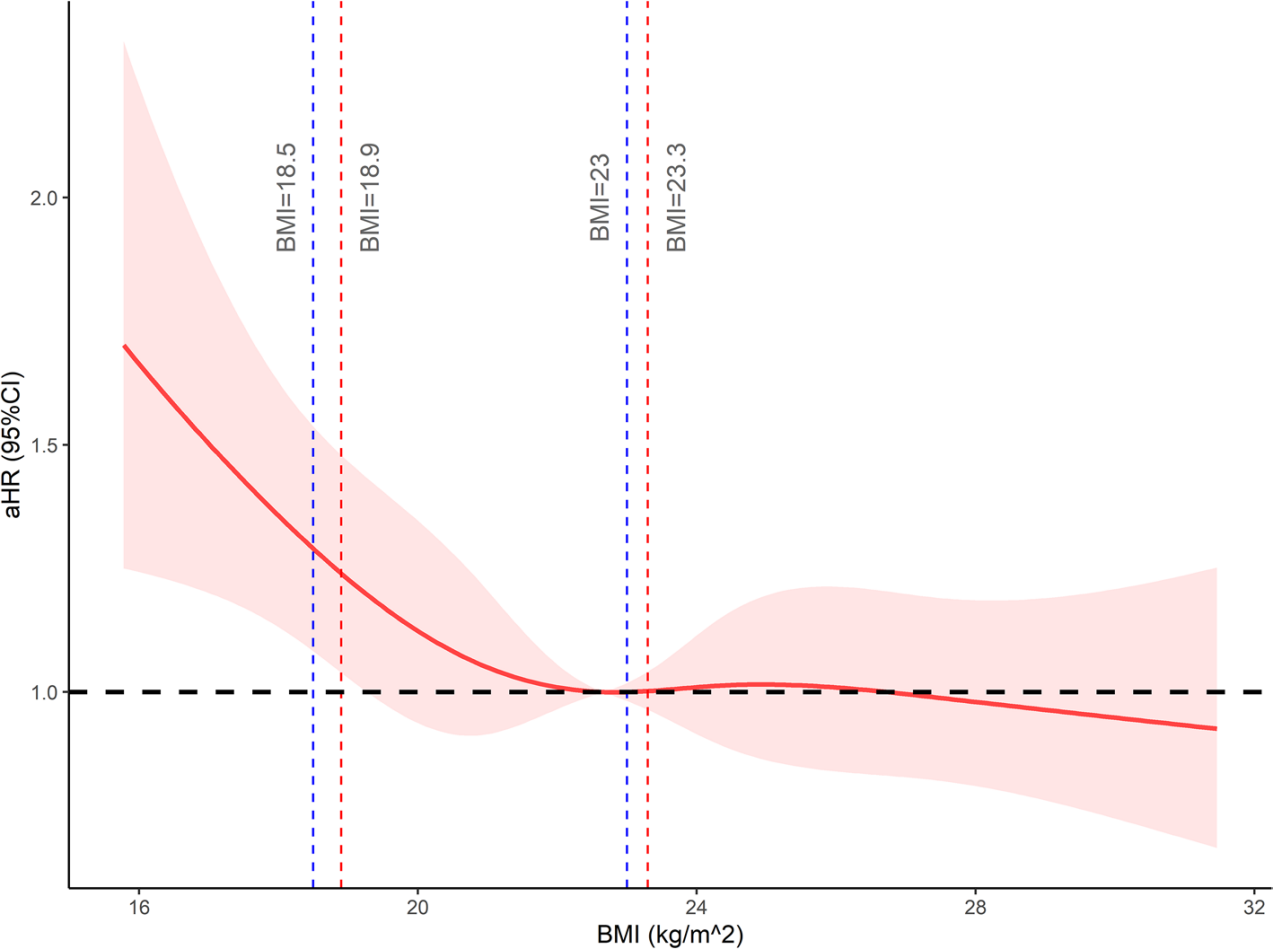
The relationship between BMI and OS was explored using RCS function based on the Cox model over 5 knots. 95% confidence bands are displayed

The association between BMI at diagnosis and the OS was depicted as RCS curves adjusted by the Cox model. (Fig. 1). The *P* values of the total correlation and nonlinear correlation were 0.004 and 0.124, respectively, suggesting that there existed a negatively correlated linear relationship between BMI and the adjusted hazard ratio (aHR). A sharp slope was observed at the beginning of the curve, and the curve tended to be smooth when BMI exceeded approximately 23 kg/m^2^.

### BMI was a positive prognostic predictor in multivariable risk adjustment analysis

As continuous data, BMI was tested fitting the PH assumption. (Pearson relevance: 0.036, *P* = 0.198) (Supp. Figure 2) In the univariate Cox survival analysis, BMI was independently analyzed as continuous data, X-tile categorical data and WHO categorical data. BMI was significantly related with the OS in all analyses. (Table 2) (X-tile: hazard ratio (HR) (overweight vs. normal) = 0.892, *P* = 0.058; WHO: HR (overweight vs. normal) = 0.901, *P* = 0.076).

**Table 2:** Univariate analysis of risks of OS

	Univariate	
	HR	*P* value
Age	1.012 (1.006–1.018)	<0.001
Male	1.044 (0.932–1.170)	0.453
ALB	0.991 (0.981–1.002)	0.106
FBG	0.998 (0.992–1.004)	0.59
logCA199	1.318 (1.236–1.406)	<0.001
TB	1.000 (1.000–1.001)	0.164
Biliary drainage	1.057 (0.929–1.203)	0.398
Body/tail	1.085 (0.971–1.212)	0.149
Diagnostic year		0.103
2013	Ref.	
2014	1.029 (0.762–1.389)	0.852
2015	1.204 (0.910–1.594)	0.194
2016	0.937 (0.715–1.228)	0.637
2017	1.120 (0.858–1.460)	0.405
2018	1.153 (0.880–1.512)	0.301
2019	0.960 (0.707–1.304)	0.795
ASA Score		0.798
1	Ref.	
2	0.977 (0.867–1.102)	0.709
3	0.926 (0.748–1.148)	0.485
4	1.180 (0.707–1.968)	0.527
Stage		<0.001
Ia	Ref.	
Ib	1.168 (1.508–1.946)	0.002
IIa	1.618 (1.186–2.207)	0.002
IIb	2.197 (1.717–2.811)	<0.001
III	3.213 (2.512–4.110)	<0.001
IV	6.307 (4.831–8.235)	<0.001
Differentiation		<0.001
I	Ref.	
II	2.645 (0.372–18.835)	0.331
III	3.941 (0.554–28.008)	0.171
IV	41.974 (2.616–673.418)	0.008
Unknown	1.399 (0.127–15.446)	0.784
Chemotherapy	0.511 (0.458–0.570)	<0.001
Surgical intervention	0.254 (0.207–0.309)	<0.001
BMI	0.964 (0.946–0.983)	<0.001
X-Tile categorizing		<0.001
< 18.9	Ref.	
18.9–23.3	0.745 (0.626–0.886)	0.001
≥ 23.3	0.665 (0.556–0.794)	<0.001
WHO categorizing		<0.001
< 18.5	Ref.	
18.5–23	0.625 (0.514–0.759)	<0.001
≥ 23	0.563 (0.462–0.685)	<0.001

*ALB* Albumin, *FBG* Fasten blood glucose, *TB* Total bilirubin, *HR* Hazard ratio, *Ref* Reference

**Fig. 2:**
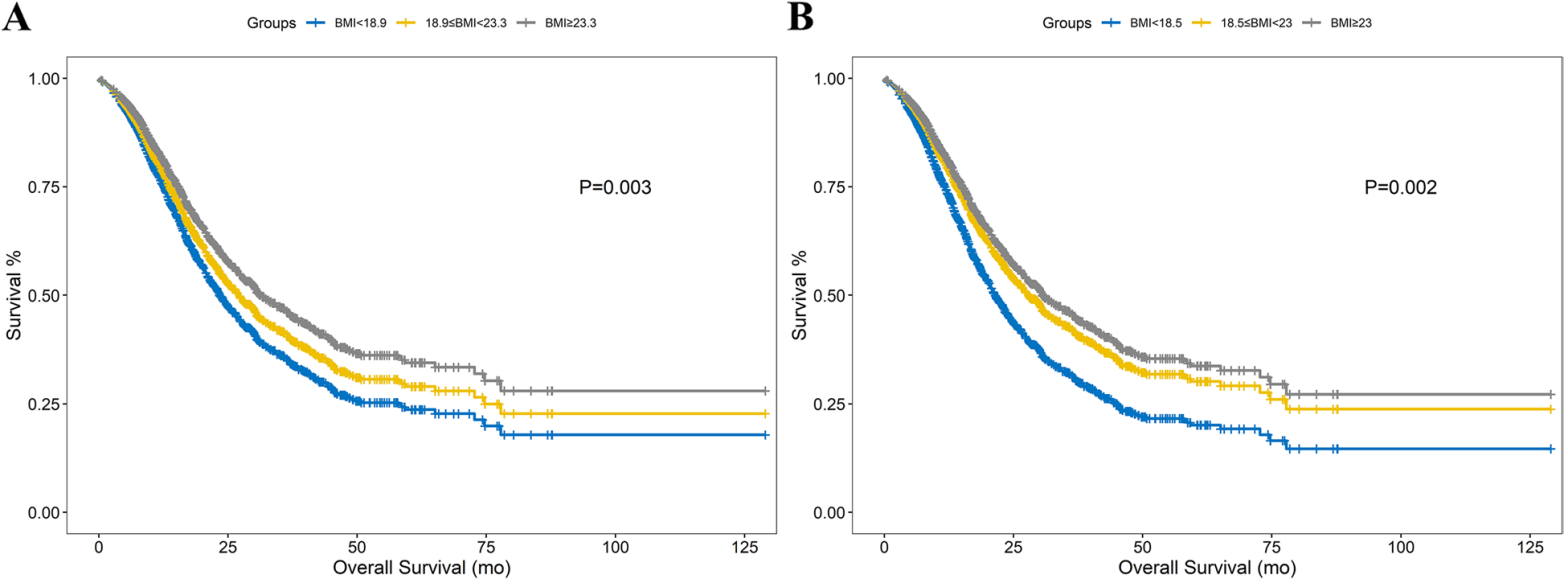
**A** Adjusted survivorship curves of the Cox model with X-tile categorical BMI. **B** The adjusted survivorship curves of the Cox model with WHO categorical BMI

Multivariable Cox regression models were established based on continuous, X-tile categorical and WHO categorical BMI data. (Fig. 2, Table 3) (X-tile: aHR (overweight vs. normal) = 0.880, *P* = 0.045; WHO: aHR (overweight vs. normal) = 0.903, *P* = 0.102) TNM stages, differentiation, CA199 and chemotherapy were significantly related to the OS in all Cox models. Both continuous and categorical BMI were discovered to be associated with chemotherapy (continuous BMI: *association P* < 0.001, X-tile categorical BMI: *association P* < 0.001, WHO categorical BMI: *association P* < 0.001). Moreover, the product term of categorical BMI and chemotherapy was significantly relevant to the OS (*interaction P*_*Xtile*_ = 0.004; *interaction P*_*WHO*_ = 0.020), suggesting the multiplicative *interactions* in the models. The additive *interaction* between BMI and chemotherapy was explored and confirmed (RERI_Xtile_: 0.50 (0.30–0.70), AP_Xtile_: 0.58 (0.29–0.86), S_Xtile_: 0.21 (0.06–0.75); RERI_WHO_: 0.56 (0.37–0.74), AP_WHO_: 0.70 (0.37–1.02), S_WHO_: 0.26 (0.12–0.57)). Nevertheless, neither *association* (Table 1, Supp. Table 1, *P*_Xtile_ = 0.162, *P*_WHO_ = 0.099) nor *interaction* (*P*_Xtile_ = 0.699, *P*_WHO_ = 0.966) was observed between categorical BMI and TNM stage.

**Table 3:** Multivariate analyses of risks of OS

	Continuous BMI		WHO Model		X-tile Model	
	aHR	*P* value	aHR	*P* value	aHR	*P* value
Age	1.004 (0.998–1.011)	0.223	1.004 (0.997–1.01)	0.279	1.004 (0.997–1.011)	0.234
Male (female as Ref.)	1.120 (0.992–1.266)	0.068	1.124 (0.995–1.271)	0.060	1.122 (0.993–1.268)	0.064
ALB	1.004 (0.992–1.017)	0.51	1.004 (0.992–1.016)	0.541	1.005 (0.993–1.017)	0.43
FBG	1.018 (0.997–1.039)	0.086	1.017 (0.996–1.038)	0.111	1.018 (0.998–1.039)	0.084
logCA199	1.164 (1.093–1.240)	<0.001	1.161 (1.09–1.236)	<0.001	1.162 (1.091–1.237)	<0.001
TB	1.000 (0.999–1.001)	0.986	1 (0.999–1.001)	0.902	1 (0.999–1.001)	0.899
Biliary drainage (no as Ref.)	1.113 (0.941–1.316)	0.213	1.109 (0.938–1.311)	0.227	1.114 (0.942–1.318)	0.205
ASA Score		0.142		0.163		0.151
1	Ref.		Ref.		Ref.	
2	0.926 (0.817–1.050)	0.231	0.926 (0.817–1.05)	0.231	0.927 (0.818–1.051)	0.239
3	1.182 (0.934–1.495)	0.164	1.179 (0.931–1.493)	0.171	1.184 (0.935–1.498)	0.16
4	1.317 (0.753–2.305)	0.334	1.272 (0.727–2.228)	0.400	1.298 (0.742–2.271)	0.36
Stage		<0.001		<0.001		<0.001
Ia	Ref.		Ref.		Ref.	
Ib	1.587 (1.210–2.080)	0.001	1.58 (1.205–2.072)	0.001	1.574 (1.2–2.064)	0.001
IIa	1.704 (1.226–2.370)	0.002	1.681 (1.209–2.337)	0.002	1.691 (1.216–2.351)	0.002
IIb	2.399 (1.846–3.117)	<0.001	2.384 (1.834–3.098)	<0.001	2.391 (1.84–3.107)	<0.001
III	3.568 (2.744–4.640)	<0.001	3.542 (2.724–4.607)	<0.001	3.558 (2.736–4.627)	<0.001
IV	6.478 (4.856–8.641)	<0.001	6.442 (4.831–8.591)	<0.001	6.501 (4.874–8.671)	<0.001
Differentiation		<0.001		<0.001		<0.001
I	Ref.		Ref.		Ref.	
II	4.642 (0.649–33.205)	0.126	4.725 (0.66–33.815)	0.122	4.762 (0.665–34.092)	0.12
III	6.444 (0.903–46.008)	0.063	6.562 (0.918–46.882)	0.061	6.637 (0.929–47.436)	0.059
IV	\		\		\	\
Unknown	1.652 (0.147–18.599)	0.684	1.744 (0.155–19.613)	0.652	1.744 (0.155–19.61)	0.652
Chemotherapy	0.458 (0.405–0.518)	<0.001	0.462 (0.408–0.522)	<0.001	0.462 (0.408–0.522)	<0.001
BMI	0.972 (0.953–0.991	0.004				
BMI Stage				0.002		0.003
Underweight			Ref.		Ref.	
Normal			0.759 (0.617–0.935)	0.010	0.821 (0.682–0.989)	0.038
Overweight			0.685 (0.555–0.847)	0.000	0.723 (0.597–0.875)	0.001

*ALB* Albumin, *FBG* Fasten blood glucose, *TB* Total bilirubin, *aHR* Adjusted hazard ratio, *Ref* Reference

### Patients with higher BMI gain more OS benefits from chemotherapy

In the different BMI categories, the ratio of patients receiving chemotherapy differed significantly. (Underweight_Xtile_: 43.1%, Normal_Xtile_: 57.0%, Overweight_Xtile_: 60.5%, *P* < 0.001; Underweight_WHO_: 40.8%, Normal_WHO_: 56.0%, Overweight_WHO_: 60.7%, *P* < 0.001). In different Cox models stratified by categorical BMI, chemotherapy had different impacts on prognosis, indicating that the patients with higher BMI benefitted more from chemotherapy. (aHR: Underweight_Xtile_: 0.565 (0.389–0.819), Normal_Xtile_: 0.474 (0.395–0.567), Overweight_Xtile_: 0.409 (0.337–0.496); Underweight_WHO_: 0.613 (0.401–0.940), Normal_WHO_: 0.464 (0.387–0.557), Overweight_WHO_: 0.425 (0.353–0.512)). Due to different chemotherapeutic strategies between patients with resectable and advanced disease, stratified by categorical BMI and resectability, we further confirmed the improved chemotherapy effect in patients with a higher BMI. (Supp. Table 2) Stratified by chemotherapy administration, it was discovered that categorical BMI was no longer associated with the OS in patients who did not receive chemotherapy in the multivariable Cox models. (Supp. Table 3, Supp. Table 4).

### Propensity score analysis revealed the role of BMI additionally

**Fig. 3:**
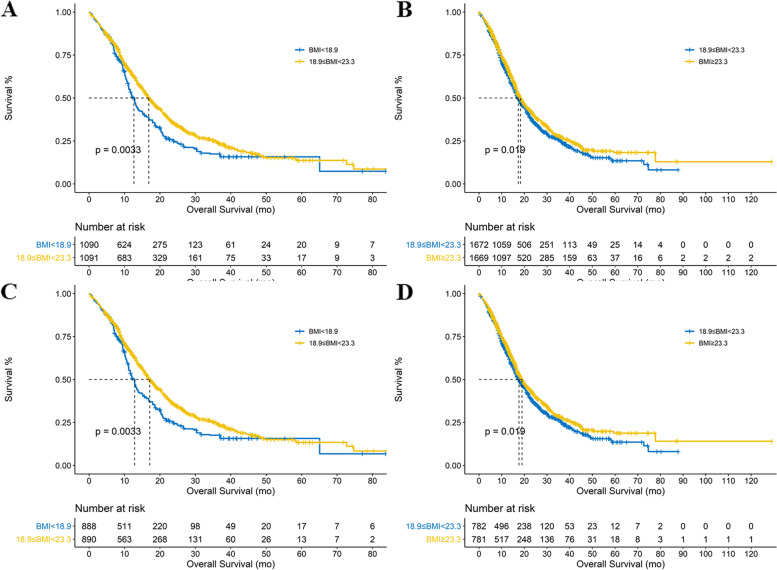
A Kaplan-Meier curves of the Underweight_Xtile_ group and Normal_Xtile_ group in the IPTW cohort. **B** Kaplan-Meier curves of Overweight_Xtile_ group and Normal_Xtile_ group in the IPTW cohort. **C** Kaplan-Meier curves of the Underweight_Xtile_ group and Normal_Xtile_ group in the SMRW cohort. **D** Kaplan-Meier curves of Overweight_Xtile_ group and Normal_Xtile_ group in the SMRW cohort

**Fig. 4:**
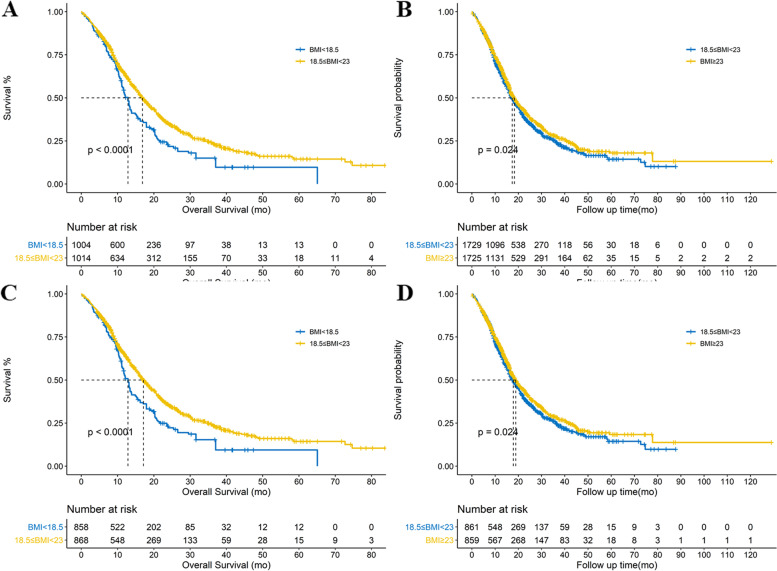
**A** Kaplan-Meier curves of the Underweight_WHO_ group and Normal_WHO_ group in the IPTW cohort. **B** Kaplan-Meier curves of Overweight_WHO_ group and Normal_WHO_ group in the IPTW cohort. **C** The Kaplan-Meier curves of the Underweight_WHO_ group and Normal_WHO_ group in the SMRW cohort. **D** Kaplan-Meier curves of Overweight_WHO_ group and Normal_WHO_ group in the SMRW cohort

IPTW and SMRW analyses were performed not only between the underweight category and normal category but also between the overweight category and normal category. The characteristics of the weighted cohorts were shown. (Table 4, Supp. Table 5, Supp. Table 6, Supp. Table 7) BMI was still an independent prognostic predictor with balanced chemotherapy status. (Fig. 3, Fig. 4).

**Table 4:** Baseline data comparisons after IPTW analysis. (categorized by X-tile cutoffs)

	Underweight (1089.88)	Normal (1091.45)	*P* value	SMD	Normal (1672.01)	Overweight (1668.85)	*P* value	SMD
Age	64 (58–70)	63 (58–70)	0.534	0.055	63.00 (57.77–69.00)	64.00 (57.00–69.00)	0.982	0.001
Female (%)	463.5 (42.5)	451.5 (41.4)	0.783	0.023	615.5 (36.8)	613.3 (36.7)	0.978	0.001
Asa (%)			1	0.01			1	0.003
1	631.8 (58.0)	635.6 (58.2)			934.9 (55.9)	934.5 (56.0)		
2	383.3 (35.2)	379.3 (34.8)			599.6 (35.9)	597.6 (35.8)		
3	65.0 (6.0)	66.5 (6.1)			123.3 (7.4)	122.3 (7.3)		
4	9.8 (0.9)	10.0 (0.9)			14.3 (0.9)	14.5 (0.9)		
Differentiation (%)			0.266	0.109			0.985	0.009
I	0.0 (0.0)	6.0 (0.5)			7.0 (0.4)	6.1 (0.4)		
II	345.2 (31.7)	358.7 (32.9)			534.5 (32.0)	531.6 (31.9)		
III	744.7 (68.3)	726.7 (66.6)			1130.5 (67.6)	1131.2 (67.8)		
Stage (%)			0.989	0.064			1	0.003
Ia	97.7 (9.0)	98.4 (9.0)			162.2 (9.7)	162.6 (9.7)		
Ib	237.0 (21.7)	247.4 (22.7)			401.2 (24.0)	401.3 (24.0)		
IIa	86.1 (7.9)	85.2 (7.8)			128.6 (7.7)	128.6 (7.7)		
IIb	256.9 (23.6)	272.3 (24.9)			431.5 (25.8)	431.3 (25.8)		
III	269.5 (24.7)	265.5 (24.3)			359.5 (21.5)	357.6 (21.4)		
IV	142.7 (13.1)	122.6 (11.2)			188.9 (11.3)	187.5 (11.2)		
Biliary drainage (%)	188.1 (17.3)	179.0 (16.4)	0.785	0.023	275.4 (16.5)	274.0 (16.4)	0.978	0.001
TB	17.10 (11.36–65.50)	16.60 (11.20–66.31)	0.675	0.018	16.70 (11.22–65.63)	16.33 (11.70–66.45)	0.989	0.001
AIB	39.00 (36.00–42.00)	39.00 (36.00–42.00)	0.711	0.011	39.00 (37.00–42.00)	39.00 (36.00–43.00)	0.958	0.003
FBG	5.82 (5.08–7.26)	5.99 (5.30–7.35)	0.489	0.065	6.06 (5.36–7.43)	6.14 (5.45–7.55)	1	< 0.001
chemotherapy (%)	587.3 (53.9)	598.8 (54.9)	0.816	0.02	989.8 (59.2)	990.0 (59.3)	0.959	0.003
CA199	167.53 (41.57–736.56)	152.50 (39.30–481.59)	0.225	0.016	149.90 (39.30–465.31)	164.78 (41.49–561.24)	0.925	0.005

*ALB* Albumin, *FBG* Fasten blood glucose, *TB* Total bilirubin, *SMD* Standard deviation mean difference

## Discussion

BMI, a simple, inexpensive and readily available parameter, has been broadly applied in the evaluation of diseases, such as metabolic, endocrine, and oncological diseases [20–24]. The role of BMI in pancreatic cancer oncogenesis has been previously studied and discussed. Higher BMI is related to an increased incidence of pancreatic cancer [3, 4]. But for patients diagnosed with pancreatic cancer, how BMI influences their prognoses is still unclear and controversial.

Okura T. et al. and Tsai S. et al. reported the positive impact of high BMI on PDAC patients’ prognoses [9, 25]. However, the sample size of Dr. Okura T.’s work was limited. In Dr. Tsai’s work, researchers analyzed the OS and BMI using Cox models without chemotherapy parameter included. Li D., Yuan C. and McWilliams R.R. held different opinions [26–28]. In Dr. McWilliams’ research, the study cohort was characterized by the advanced stage and high average BMI (stage III&IV: 69.9%, BMI: 27.6 (24.4–31.2) kg/m^2^, BMI < 18.5: 0.7%). In addition, chemotherapy administration was not discussed or analyzed. In the studies of Dr. Li and Dr. Yuan, the early-onset pancreatic cancer pattern [29], the lack of adjuvant/neoadjuvant treatment data and the baseline data differences might explain the differences between their results and ours. In some studies, it was proposed that no association existed between BMI and the OS [10, 30–32]. Sample size, covariates included in the studies and racial/regional differences might lead to conclusion divergence. Above all, the differences between previous works and ours could be explained by baseline data differences (e.g., racial/regional background, TNM stages), the covariates included and the sample size. In addition, the advancement of chemotherapy is another possible cause for the conclusion divergence due to the vital role of chemotherapy in the protective impact of BMI on the OS .

In the RCS analysis, a negative linear relationship between BMI and aHR was observed. There was a strong association with a higher risk of death in underweight patients than in normal weight individuals. The curve tends to be smooth when BMI is above ~ 23 kg/m^2^. In the univariate analysis, the tumor location and the diagnostic year were filtered out for the multivariable analysis. Categorical BMI grouped by both standards and continuous BMI were significantly associated with the OS. FBG and ALB were 2 additional parameters used to evaluate the nutrition condition. Neither the significant impact on the OS (Table 3) nor the correlation with BMI (FBG: *P* = 0.087, ALB: *P* = 0.060) was observed in this study.

Chemotherapy was discovered to be related to both continuous BMI and categorical BMI. No significant *association* or *interaction* was observed between TNM stage and BMI. More patients were treated with chemotherapy in the higher BMI groups. Stratified by BMI level, we compared the impact of chemotherapy in different categories. Chemotherapy had a more positive impact on the OS in the higher BMI categories. After further stratification by different chemotherapy strategies, a similar phenomenon was still observed. Stratified by chemotherapy administrated or not, the protective impact of BMI was only observed in the chemotherapy-administered subcategory. Above all, we discovered that higher BMI might prolong the OS time on the basis of chemotherapy. And we hypothesized that this protective effect was probably via the improved response to chemotherapy.

Given that there existed an *interaction* and *association* between BMI and chemotherapy, IPTW analysis was then introduced as a sensitivity analysis. With pairwise comparisons, compared with normal-weight patients, being underweight was discovered to be a risk factor, while being overweight was a protective factor. Provided that the chemotherapy acceptance was matched in different categories, the results furtherly support our hypothesis that higher BMI conferred patients better responses to chemotherapy. The consistency of the IPTW and SMRW analysis results indicated that no significant confounding/interacting factor was excluded.

Some limitations in this study should be stressed. First, though PS analysis and multivariable risk adjustment were performed, the retrospective single-center data still limited the reliability and extrapolation of this study. A multicenter prospective study analyzing BMI could help validate the results. Second, given that all enrolled subjects were from the Chinese Han population, further research on patients from different districts and races should be conducted. Additionally, the duration and courses of chemotherapy were not listed and analyzed. Sufficient or insufficient chemotherapy courses might lead to different outcomes, and how BMI influences these outcomes was undefined. Finally, selection bias may be present due to the preference for patients with resectable disease at our pancreatic disease center. However, with multivariate, PS and stratified analyses, we sought to fix the bias for reliable conclusions.

In this study, we elaborated on the relationship between BMI and the OS of PDAC patients based on IPTW, RCS and multivariable risk adjustment analyses and shed light on the probable underlying mechanism. Higher BMI indicated more frequent chemotherapy treatment and longer OS among PDAC patients. Among the different BMI categories, patients with a higher BMI also benefitted more from chemotherapy. The protective impact of high BMI disappeared when chemotherapy was not applied. Thus we hypothesized that high BMI might affect the OS of PDAC patients via the acceptance and response of chemotherapy. The relevance between BMI and chemotherapy requires further study.

## Supplementary Information

**Additional file 1: Supplementary Fig. 1.** The flow-chart of case enrollment.

**Additional file 2: Supplementary Table 1.** Demographic and baseline characteristics of study cohort. (Categorized by WHO cutoffs).

**Additional file 3: Supplementary Fig. 2.** The Schoenfeld residual plot of BMI with rank of OS for PH assumption test.

**Additional file 4: Supp. Table 2.** Chemotherapy impact on OS stratified with categorical BMI and resectability.

**Additional file 5: Supp. Table 3.** Multivariate analyses of risks of OS stratified with chemotherapy administration (categorized by Xtile cutoffs).

**Additional file 6: Supp. Table 4.** Multivariate analyses of risks of OS stratified with chemotherapy administration (categorized by WHO cutoffs).

**Additional file 7: Supplementary Table 5.** Baseline data comparisons after SMRW analysis. (categorized by Xtile cutoffs).

**Additional file 8: Supplementary Table 6.** Baseline data comparisons after IPTW analysis. (categorized by WHO cutoffs).

**Additional file 9: Supplementary Table 7.** Baseline data comparisons after SMRW analysis. (categorized by WHO cutoffs).

## Data Availability

The raw data should be inaccessible to the public. The statistical data and results are available. Data can be requested by contacting fnz01b74@rjh.com.cn.
