# Supplementary figures and images for: Higher body mass index indicated better overall survival in pancreatic ductal adenocarcinoma patients: a real-world study of 2010 patients
Source: BMC Cancer. 2021 Dec 9;21:1318. doi: 10.1186/s12885-021-09056-0 (PMC8656027; doi:10.1186/s12885-021-09056-0)

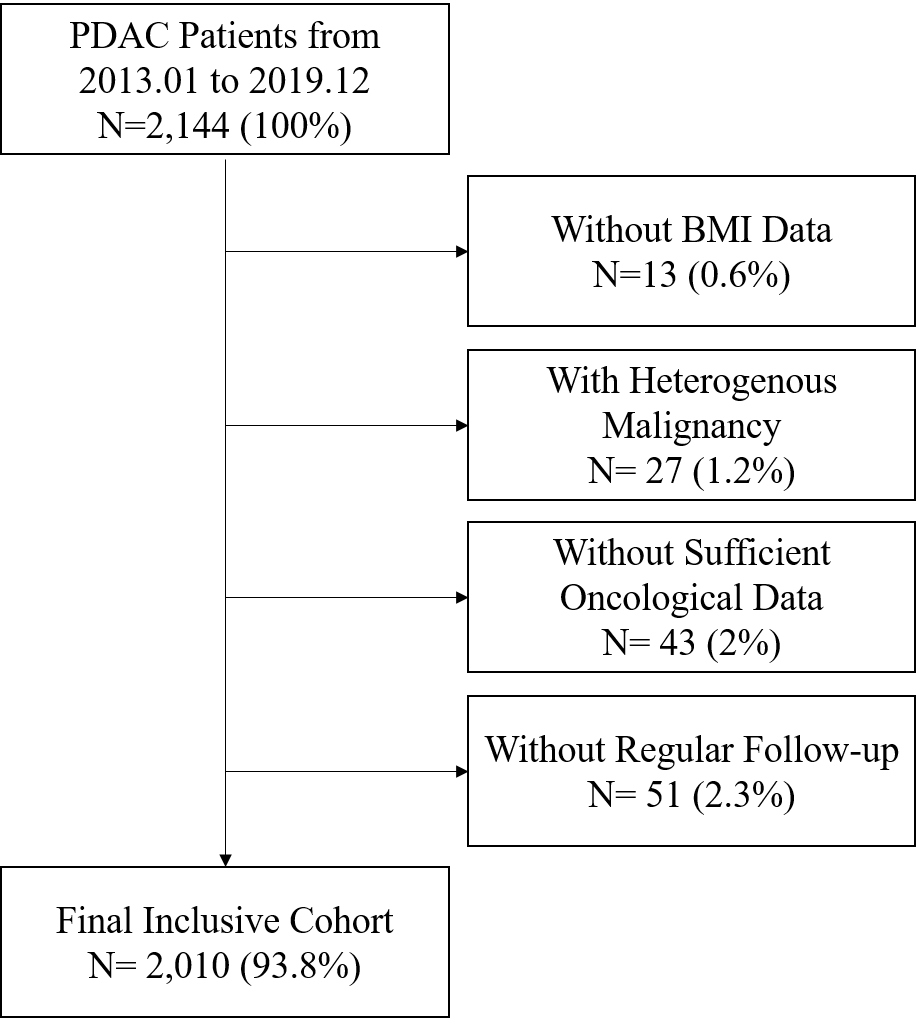


Supplementary Figure 1. The flow-chart of case enrollment.

Supplement: Supplementary file 1 — Additional file 1: Supplementary Fig. 1. The flow-chart of case enrollment. [file 12885_2021_9056_MOESM1_ESM.docx]
