# Supplementary material for: Higher body mass index indicated better overall survival in pancreatic ductal adenocarcinoma patients: a real-world study of 2010 patients
Source: BMC Cancer. 2021 Dec 9;21:1318. doi: 10.1186/s12885-021-09056-0 (PMC8656027; doi:10.1186/s12885-021-09056-0)
Supplement: Supplementary file 2 — Additional file 2: Supplementary Table 1. Demographic and baseline characteristics of study cohort. (Categorized by WHO cutoffs). [file 12885_2021_9056_MOESM2_ESM.docx]

Supplementary Table 1. Demographic and baseline characteristics of study cohort. (Categorized by WHO cutoffs)

|  | Underweight (n=157) | Normal (n=943) | Overweight (n=910) | total | P value |
| --- | --- | --- | --- | --- | --- |
| Age | 66 (60-73) | 63 (58-69) | 64 (57-69) | 63 (58-69) | 0.001 |
| Male (%) | 80 (51) | 572 (60.7) | 599 (65.8) | 1251 (62.2) | 0.001 |
| ALB | 38 (35-42) | 39 (36-42) | 40 (36-43) | 39 (36-42) | 0.083 |
| FBG | 5.79 (4.97-7.00) | 5.97 (5.30-7.31) | 6.10 (5.41-7.56) | 6.03 (5.32-7.40) | 0.009 |
| CA199 | 217.6 (34.8-896.3) | 152.6 (40.0-492.5) | 162.8 (41.0-550.7) | 161.1 (40.2-552.2) | 0.303 |
| TB | 16.8 (11.7-68.9) | 16.8 (11.3-70.4) | 16.7 (11.9-75.4) | 16.8 (11.6-72.6) | 0.792 |
| Biliary drainage (%) | 43 (27.4) | 189 (20) | 203 (22.3) | 324 (21.6) | 0.095 |
| ASA Score (%) |  |  |  |  | 0.281 |
| 1 | 94 (61.4) | 526 (57.5) | 473 (53.5) | 1093 (56) |  |
| 2 | 48 (31.4) | 316 (34.5) | 332 (37.6) | 696 (35.7) |  |
| 3 | 9 (5.9) | 62 (6.8) | 73 (8.3) | 144 (7.4) |  |
| 4 | 2 (1.3) | 11 (1.2) | 6 (0.7) | 19 (1) |  |
| Tumor location (%) |  |  |  |  | 0.827 |
| Head | 90 (57.3) | 528 (56) | 522 (57.4) | 1140 (56.7) |  |
| Body/Tail | 67 (42.7) | 415 (44) | 388 (42.6) | 870 (43.3) |  |
| Diagnostic year (%) |  |  |  |  | 0.608 |
| 2013 | 4 (2.5) | 38 (4) | 34 (3.7) | 76 (3.8) |  |
| 2014 | 16 (10.2) | 53 (5.6) | 63 (6.9) | 132 (6.6) |  |
| 2015 | 16 (10.2) | 104 (11) | 87 (9.6) | 207 (10.3) |  |
| 2016 | 21 (13.4) | 146 (15.5) | 146 (16) | 313 (15.6) |  |
| 2017 | 33 (21) | 195 (20.7) | 168 (18.5) | 396 (19.7) |  |
| 2018 | 36 (22.9) | 198 (21) | 192 (21.1) | 426 (21.2) |  |
| 2019 | 31 (19.7) | 209 (22.2) | 220 (24.2) | 460 (22.9) |  |
| Stage |  |  |  |  | 0.099 |
| Ia | 12 (7.6) | 84 (8.9) | 96 (10.5) | 192 (9.6) |  |
| Ib | 35 (22.3) | 213 (22.6) | 230 (25.3) | 478 (23.8) |  |
| IIa | 15 (9.6) | 71 (7.5) | 69 (7.6) | 155 (7.7) |  |
| IIb | 37 (23.6) | 249 (26.4) | 246 (27) | 532 (26.5) |  |
| III | 37 (23.6) | 231 (24.5) | 164 (18) | 432 (21.5) |  |
| IV | 21 (13.4) | 95 (10.1) | 105 (11.5) | 221 (11) |  |
| Differentiation (%) |  |  |  |  | 0.323 |
| I | 0 (0) | 4 (0.4) | 3 (0.3) | 7 (0.3) |  |
| II | 45 (28.7) | 324 (34.4) | 281 (30.9) | 650 (32.3) |  |
| III | 112 (71.3) | 611 (64.8) | 626 (68.8) | 1349 (67.1) |  |
| IV | 0 (0) | 1 (0.1) | 0 (0) | 1 (0) |  |
| Unknown | 0 (0) | 3 (0.3) | 0 (0) | 3 (0.1) |  |
| Chemotherapy (%) | 64 (40.8) | 528 (56) | 552 (60.7) | 1144 (56.9) | <0.001 |
| Surgical interventions (%) | 111 (70.7) | 659 (69.9) | 649 (71.3) | 1419 (70.6) | 0.649 |
| ALB, albumin; FBG, fasten blood glucose; TB, total bilirubin. | | | | | |
