# Supplementary material for: Higher body mass index indicated better overall survival in pancreatic ductal adenocarcinoma patients: a real-world study of 2010 patients
Source: BMC Cancer. 2021 Dec 9;21:1318. doi: 10.1186/s12885-021-09056-0 (PMC8656027; doi:10.1186/s12885-021-09056-0)
Supplement: Supplementary file 3 — Additional file 3: Supplementary Fig. 2. The Schoenfeld residual plot of BMI with rank of OS for PH assumption test. [file 12885_2021_9056_MOESM3_ESM.docx]

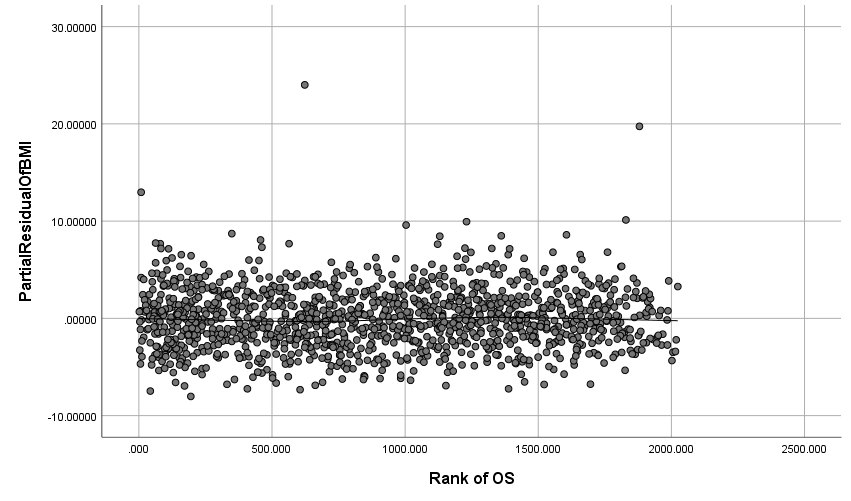


Supplementary Figure 2. The Schoenfeld residual plot of BMI with rank of OS for PH assumption test.
