# Supplementary material for: Higher body mass index indicated better overall survival in pancreatic ductal adenocarcinoma patients: a real-world study of 2010 patients
Source: BMC Cancer. 2021 Dec 9;21:1318. doi: 10.1186/s12885-021-09056-0 (PMC8656027; doi:10.1186/s12885-021-09056-0)
Supplement: Supplementary file 4 — Additional file 4: Supp. Table 2. Chemotherapy impact on OS stratified with categorical BMI and resectability. [file 12885_2021_9056_MOESM4_ESM.docx]

Supp. Table 2. Chemotherapy impact on OS stratified with categorical BMI and resectability.

|  | Resectable | | Advanced | |
| --- | --- | --- | --- | --- |
|  | aHR | P value | aHR | P value |
| Underweight_WHO_ | 0.750 (0.445-1.265) | 0.281 | 0.497 (0.234-1.054) | 0.068 |
| Normal_WHO_ | 0.619 (0.489-0.784) | <0.001 | 0.348 (0.264-0.460) | <0.001 |
| Overweight_WHO_ | 0.444 (0.344-0.572) | <0.001 | 0.327 (0.242-0.442) | <0.001 |
| Underweight_Xtile_ | 0.792 (0.501-1.253) | 0.320 | 0.360 (0.188-0.691) | 0.002 |
| Normal_Xtile_ | 0.597 (0.470-0.759) | <0.001 | 0.371 (0.282-0.488) | <0.001 |
| Overweight_Xtile_ | 0.428 (0.330-0.557) | <0.001 | 0.309 (0.224-0.425) | <0.001 |
| aHR, adjusted hazard ratio. | | | | |
