# Supplementary material for: Higher body mass index indicated better overall survival in pancreatic ductal adenocarcinoma patients: a real-world study of 2010 patients
Source: BMC Cancer. 2021 Dec 9;21:1318. doi: 10.1186/s12885-021-09056-0 (PMC8656027; doi:10.1186/s12885-021-09056-0)
Supplement: Supplementary file 5 — Additional file 5: Supp. Table 3. Multivariate analyses of risks of OS stratified with chemotherapy administration (categorized by Xtile cutoffs). [file 12885_2021_9056_MOESM5_ESM.docx]

Supp. Table 3. Multivariate analyses of risks of OS stratified with chemotherapy administration (categorized by Xtile cutoffs).

|  | No chemotherapy | | | Chemotherapy | | |
| --- | --- | --- | --- | --- | --- | --- |
|  | aHR | 95% CI | P value | aHR | 95% CI | P value |
| Age | 1.001 | (0.992-1.009) | 0.892 | 1.008 | (0.998-1.019) | 0.109 |
| Male | 1.212 | (1.023-1.435) | 0.026 | 1.03 | (0.861-1.233) | 0.745 |
| ASA Score | |  | 0.306 |  |  | 0.224 |
| 1 | Ref. |  |  | Ref. |  |  |
| 2 | 0.994 | (0.835-1.183) | 0.947 | 0.822 | (0.681-0.991) | 0.04 |
| 3 | 1.284 | (0.939-1.755) | 0.117 | 0.864 | (0.596-1.252) | 0.44 |
| 4 | 1.452 | (0.704-2.994) | 0.313 | 0.982 | (0.401-2.408) | 0.969 |
| logCA199 | 1.128 | (1.037-1.226) | 0.005 | 1.212 | (1.098-1.338) | <0.001 |
| TB | 1 | (0.999-1.001) | 0.695 | 1.001 | (0.999-1.002) | 0.265 |
| FBG | 1.006 | (0.978-1.035) | 0.676 | 1.03 | (1-1.059) | 0.047 |
| ALB | 1.011 | (0.993-1.029) | 0.221 | 1 | (0.983-1.017) | 0.97 |
| Differentiation | |  | <0.001 |  |  | 0.044 |
| I | Ref. |  |  | Ref. |  |  |
| II | 4.301 | (0.597-30.975) | 0.148 | 82.949 | (0-6.38E17) | 0.813 |
| III | 6.042 | (0.842-43.36) | 0.074 | 106.783 | (0-8.21E17) | 0.802 |
| IV | 1.614 | (0.141-18.436) | 0.7 |  |  |  |
| Biliary drainage | 1.218 | (0.974-1.524) | 0.084 | 0.99 | (0.767-1.277) | 0.935 |
| BMI stage |  |  | 0.287 |  |  | <0.001 |
| Underweight | Ref. |  |  | Ref. |  |  |
| Normal | 0.911 | (0.717-1.156) | 0.442 | 0.562 | (0.417-0.758) | <0.001 |
| Overweight | 0.826 | (0.645-1.06) | 0.133 | 0.458 | (0.337-0.622) | <0.001 |
| TNM Stage | |  | <0.001 |  |  | <0.001 |
| Ia | Ref. |  |  | Ref. |  |  |
| Ib | 1.443 | (0.952-2.185) | 0.084 | 1.386 | (0.885-2.17) | 0.154 |
| IIa | 1.229 | (0.797-1.897) | 0.351 | 1.258 | (0.795-1.99) | 0.327 |
| IIb | 2 | (1.346-2.971) | 0.001 | 2.181 | (1.435-3.315) | <0.001 |
| III | 3.106 | (2.082-4.633) | <0.001 | 2.905 | (1.909-4.42) | <0.001 |
| IV | 5.812 | (3.79-8.912) | <0.001 | 4.895 | (3.063-7.821) | <0.001 |
| ALB, albumin; FBG, fasten blood glucose; TB, total bilirubin; aHR, adjusted hazard ratio; CI, confidence interval; Ref., reference. | | | | | | |
