# Supplementary material for: Higher body mass index indicated better overall survival in pancreatic ductal adenocarcinoma patients: a real-world study of 2010 patients
Source: BMC Cancer. 2021 Dec 9;21:1318. doi: 10.1186/s12885-021-09056-0 (PMC8656027; doi:10.1186/s12885-021-09056-0)
Supplement: Supplementary file 6 — Additional file 6: Supp. Table 4. Multivariate analyses of risks of OS stratified with chemotherapy administration (categorized by WHO cutoffs). [file 12885_2021_9056_MOESM6_ESM.docx]

Supp. Table 4. Multivariate analyses of risks of OS stratified with chemotherapy administration (categorized by WHO cutoffs).

|  | No chemotherapy | | | Chemotherapy | | |
| --- | --- | --- | --- | --- | --- | --- |
|  | aHR | 95% CI | P value | aHR | 95% CI | P value |
| Age | 1 | (0.992-1.009) | 0.922 | 1.006 | (0.996-1.017) | 0.237 |
| Male | 1.214 | (1.025-1.438) | 0.025 | 1.019 | (0.851-1.219) | 0.839 |
| ASA Score | |  | 0.311 |  |  | 0.253 |
| 1 | Ref. |  |  | Ref. |  |  |
| 2 | 0.993 | (0.835-1.182) | 0.94 | 0.83 | (0.688-1.001) | 0.051 |
| 3 | 1.282 | (0.938-1.754) | 0.119 | 0.845 | (0.582-1.226) | 0.375 |
| 4 | 1.443 | (0.7-2.977) | 0.321 | 0.929 | (0.378-2.285) | 0.873 |
| logCA199 | 1.128 | (1.038-1.226) | 0.005 | 1.204 | (1.091-1.328) | <0.001 |
| TB | 1 | (0.999-1.001) | 0.688 | 1.001 | (0.999-1.002) | 0.236 |
| FBG | 1.005 | (0.977-1.034) | 0.745 | 1.028 | (0.998-1.058) | 0.064 |
| ALB | 1.01 | (0.993-1.028) | 0.247 | 0.996 | (0.979-1.014) | 0.68 |
| Differentiation | |  | <0.001 |  |  | 0.061 |
| I | Ref. |  |  | Ref. |  |  |
| II | 4.235 | (0.588-30.48) | 0.152 | 80.655 | (0-7.40E17) | 0.815 |
| III | 5.933 | (0.827-42.55) | 0.077 | 102.437 | (0-9.40E17) | 0.805 |
| IV | 1.606 | (0.141-18.34) | 0.703 |  |  |  |
| Biliary drainage | 1.217 | (0.973-1.522) | 0.085 | 0.964 | (0.746-1.245) | 0.778 |
| BMI stage |  |  | 0.195 |  |  | <0.001 |
| Underweight | Ref. |  |  | Ref. |  |  |
| Normal | 0.835 | (0.643-1.084) | 0.175 | 0.488 | (0.343-0.692) | <0.001 |
| Overweight | 0.782 | (0.599-1.021) | 0.071 | 0.416 | (0.293-0.592) | <0.001 |
| TNM Stage | |  | <0.001 |  |  | <0.001 |
| Ia | Ref. |  |  | Ref. |  |  |
| Ib | 1.446 | (0.955-2.19) | 0.082 | 1.396 | (0.891-2.187) | 0.145 |
| IIa | 1.221 | (0.791-1.885) | 0.366 | 1.272 | (0.803-2.015) | 0.305 |
| IIb | 1.995 | (1.342-2.965) | 0.001 | 2.167 | (1.425-3.294) | <0.001 |
| III | 3.088 | (2.071-4.606) | <0.001 | 2.921 | (1.918-4.447) | <0.001 |
| IV | 5.766 | (3.761-8.84) | <0.001 | 4.823 | (3.018-7.71) | <0.001 |
| ALB, albumin; FBG, fasten blood glucose; TB, total bilirubin; aHR, adjusted hazard ratio; CI, confidence interval; Ref., reference. | | | | | | |
