# Supplementary material for: Higher body mass index indicated better overall survival in pancreatic ductal adenocarcinoma patients: a real-world study of 2010 patients
Source: BMC Cancer. 2021 Dec 9;21:1318. doi: 10.1186/s12885-021-09056-0 (PMC8656027; doi:10.1186/s12885-021-09056-0)
Supplement: Supplementary file 7 — Additional file 7: Supplementary Table 5. Baseline data comparisons after SMRW analysis. (categorized by Xtile cutoffs). [file 12885_2021_9056_MOESM7_ESM.docx]

Supplementary Table 5. Baseline data comparisons after SMRW analysis. (categorized by Xtile cutoffs)

|  | Underweight (887.88) | Normal (890) | P value | SMD | Normal (782.01) | Overweight (781) | P value | SMD |
| --- | --- | --- | --- | --- | --- | --- | --- | --- |
| Age | 63.00 (57.09-69.49) | 63.00 (57.00-69.00) | 0.494 | 0.067 | 63.00 (58.00-69.00) | 64.00 (57.00-70.00) | 0.58 | 0.008 |
| Female (%) | 374.5 (42.2) | 361.0 (40.6) | 0.71 | 0.033 | 254.5 (32.5) | 254.0 (32.5) | 0.992 | <0.001 |
| Asa (%) |  |  | 0.999 | 0.014 |  |  | 0.981 | 0.021 |
| 1 | 507.8 (57.2) | 512.0 (57.5) |  |  | 422.9 (54.1) | 422.0 (54.0) |  |  |
| 2 | 321.3 (36.2) | 317.0 (35.6) |  |  | 282.6 (36.1) | 287.0 (36.7) |  |  |
| 3 | 51.0 ( 5.7) | 53.0 ( 6.0) |  |  | 70.3 ( 9.0) | 66.0 ( 8.5) |  |  |
| 4 | 7.8 ( 0.9) | 8.0 ( 0.9) |  |  | 6.3 ( 0.8) | 6.0 ( 0.8) |  |  |
| Differentiation (%) |  |  | 0.073 | 0.122 |  |  | 0.995 | 0.005 |
| I | 0.0 ( 0.0) | 6.0 ( 0.7) |  |  | 1.0 ( 0.1) | 1.0 ( 0.1) |  |  |
| II | 282.2 (31.8) | 296.0 (33.3) |  |  | 238.5 (30.5) | 240.0 (30.7) |  |  |
| III | 605.7 (68.2) | 588.0 (66.1) |  |  | 542.5 (69.4) | 540.0 (69.1) |  |  |
| Stage (%) |  |  | 0.971 | 0.083 |  |  | 1 | 0.01 |
| Ia | 80.7 ( 9.1) | 81.0 ( 9.1) |  |  | 81.2 (10.4) | 82.0 (10.5) |  |  |
| Ib | 192.0 (21.6) | 203.0 (22.8) |  |  | 198.2 (25.3) | 195.0 (25.0) |  |  |
| IIa | 68.1 ( 7.7) | 67.0 ( 7.5) |  |  | 61.6 ( 7.9) | 61.0 ( 7.8) |  |  |
| IIb | 202.9 (22.9) | 220.0 (24.7) |  |  | 211.5 (27.0) | 212.0 (27.1) |  |  |
| III | 225.5 (25.4) | 221.0 (24.8) |  |  | 138.5 (17.7) | 140.0 (17.9) |  |  |
| IV | 118.7 (13.4) | 98.0 (11.0) |  |  | 90.9 (11.6) | 91.0 (11.7) |  |  |
| Biliary drainage (%) | 151.1 (17.0) | 142.0 (16.0) | 0.744 | 0.029 | 133.4 (17.1) | 130.0 (16.6) | 0.831 | 0.011 |
| TB | 17.10 (11.33-65.23) | 16.60 (11.20-65.40) | 0.773 | 0.029 | 16.70 (11.36-65.75) | 16.35 (11.70-66.88) | 0.853 | 0.004 |
| AIB | 39.00 (36.00-42.72) | 39.00 (36.00-42.00) | 0.661 | 0.009 | 39.00 (37.00-43.00) | 40.00 (36.00-43.00) | 0.792 | 0.011 |
| FBG | 5.93 (5.12-7.48) | 6.05 (5.34-7.41) | 0.627 | 0.08 | 6.07 (5.37-7.46) | 6.13 (5.43-7.56) | 0.365 | 0.013 |
| chemotherapy (%) | 502.3 (56.6) | 515.0 (57.9) | 0.763 | 0.026 | 474.8 (60.7) | 475.0 (60.8) | 0.964 | 0.002 |
| CA199 | 168.22 (55.52-732.77) | 151.30 (39.25-473.40) | 0.19 | 0.021 | 147.51 (39.30-448.54) | 162.90 (41.62-554.18) | 0.219 | 0.011 |
| ALB, albumin; FBG, fasten blood glucose; TB, total bilirubin; SMD, standard deviation mean difference. | | | | | | | | |
