# Supplementary material for: Higher body mass index indicated better overall survival in pancreatic ductal adenocarcinoma patients: a real-world study of 2010 patients
Source: BMC Cancer. 2021 Dec 9;21:1318. doi: 10.1186/s12885-021-09056-0 (PMC8656027; doi:10.1186/s12885-021-09056-0)
Supplement: Supplementary file 8 — Additional file 8: Supplementary Table 6. Baseline data comparisons after IPTW analysis. (categorized by WHO cutoffs). [file 12885_2021_9056_MOESM8_ESM.docx]

Supplementary Table 6. Baseline data comparisons after IPTW analysis. (categorized by WHO cutoffs)

|  | Underweight  (992.47) | Normal (1014.56) | P value | SMD | Normal (1728.87) | Overweight (1724.86) | P value | SMD |
| --- | --- | --- | --- | --- | --- | --- | --- | --- |
| Age | 63.71 (58.00-69.02) | 63.00 (58.00-70.00) | 0.847 | 0.002 | 63.00 (57.52-69.00) | 64.00 (57.00-70.00) | 0.604 | 0.002 |
| Female (%) | 411.1 (41.4) | 414.3 (40.8) | 0.903 | 0.012 | 635.7 (36.8) | 635.6 (36.8) | 0.974 | 0.002 |
| Asa (%) |  |  | 0.952 | 0.056 |  |  | 1 | 0.004 |
| 1 | 599.7 (60.4) | 598.0 (58.9) |  |  | 970.9 (56.2) | 968.8 (56.2) |  |  |
| 2 | 313.5 (31.6) | 344.4 (33.9) |  |  | 615.1 (35.6) | 614.7 (35.6) |  |  |
| 3 | 67.9 ( 6.8) | 62.1 ( 6.1) |  |  | 128.5 ( 7.4) | 126.8 ( 7.4) |  |  |
| 4 | 11.4 ( 1.1) | 10.0 ( 1.0) |  |  | 14.3 ( 0.8) | 14.6 ( 0.8) |  |  |
| Differentiation (%) |  |  | 0.14 | 0.089 |  |  | 0.997 | 0.002 |
| I | 0.0 ( 0.0) | 4.0 ( 0.4) |  |  | 6.9 ( 0.4) | 6.8 ( 0.4) |  |  |
| II | 333.4 (33.6) | 337.1 (33.2) |  |  | 554.0 (32.0) | 550.8 (31.9) |  |  |
| III | 659.1 (66.4) | 673.4 (66.4) |  |  | 1168.0 (67.6) | 1167.2 (67.7) |  |  |
| Stage (%) |  |  | 0.972 | 0.089 |  |  | 1 | 0.004 |
| Ia | 105.9 (10.7) | 90.7 ( 8.9) |  |  | 166.5 ( 9.6) | 167.0 ( 9.7) |  |  |
| Ib | 235.5 (23.7) | 228.3 (22.5) |  |  | 413.5 (23.9) | 412.0 (23.9) |  |  |
| IIa | 74.2 ( 7.5) | 79.9 ( 7.9) |  |  | 132.6 ( 7.7) | 132.7 ( 7.7) |  |  |
| IIb | 218.9 (22.1) | 254.6 (25.1) |  |  | 452.2 (26.2) | 453.1 (26.3) |  |  |
| III | 244.1 (24.6) | 248.0 (24.4) |  |  | 369.3 (21.4) | 367.2 (21.3) |  |  |
| IV | 113.8 (11.5) | 113.0 (11.1) |  |  | 194.7 (11.3) | 192.8 (11.2) |  |  |
| Biliary drainage (%) | 173.2 (17.5) | 164.7 (16.2) | 0.719 | 0.033 | 282.7 (16.4) | 280.5 (16.3) | 0.96 | 0.002 |
| TB | 16.28 (11.30-65.50) | 16.60 (11.20-65.69) | 0.817 | 0.005 | 16.70 (11.26-65.87) | 16.35 (11.70-67.07) | 0.809 | 0.001 |
| AIB | 38.00 (36.00-42.00) | 39.00 (36.00-42.00) | 0.274 | 0.088 | 39.00 (36.00-42.00) | 39.00 (36.00-42.00) | 0.79 | 0.003 |
| FBG | 5.82 (4.97-7.24) | 5.97 (5.29-7.30) | 0.409 | 0.014 | 5.99 (5.32-7.38) | 6.13 (5.43-7.56) | 0.101 | 0.001 |
| chemotherapy (%) | 513.3 (51.7) | 550.4 (54.3) | 0.602 | 0.051 | 1018.0 (58.9) | 1017.3 (59.0) | 0.967 | 0.002 |
| CA199 | 216.71 (32.05-841.50) | 153.30 (40.24-502.70) | 0.221 | 0.01 | 150.48 (40.11-467.27) | 162.90 (40.97-550.90) | 0.373 | 0.003 |
| ALB, albumin; FBG, fasten blood glucose; TB, total bilirubin; SMD, standard deviation mean difference. | | | | | | | | |
