# Supplementary material for: Higher body mass index indicated better overall survival in pancreatic ductal adenocarcinoma patients: a real-world study of 2010 patients
Source: BMC Cancer. 2021 Dec 9;21:1318. doi: 10.1186/s12885-021-09056-0 (PMC8656027; doi:10.1186/s12885-021-09056-0)
Supplement: Supplementary file 9 — Additional file 9: Supplementary Table 7. Baseline data comparisons after SMRW analysis. (categorized by WHO cutoffs). [file 12885_2021_9056_MOESM9_ESM.docx]

Supplementary Table 7. Baseline data comparisons after SMRW analysis. (categorized by WHO cutoffs)

|  | Underweight  (846.47) | Normal  (868) | P value | SMD | Normal (860.87) | Overweight (859) | P value | SMD |
| --- | --- | --- | --- | --- | --- | --- | --- | --- |
| Age | 63.00 (57.00-69.00) | 63.00 (58.00-69.00) | 0.834 | 0.003 | 63.00 (57.00- 69.00) | 64.00 (57.00- 70.00) | t | 0.011 |
| Female (%) | 342.1 (40.4) | 345.0 (39.7) | 0.89 | 0.014 | 290.7 (33.8) | 290.0 (33.8) | 0.996 | <0.001 |
| Asa (%) |  |  | 0.925 | 0.067 |  |  | 0.981 | 0.021 |
| 1 | 510.7 (60.3) | 508.0 (58.5) |  |  | 462.9 (53.8) | 461.0 (53.7) |  |  |
| 2 | 267.5 (31.6) | 299.0 (34.4) |  |  | 316.1 (36.7) | 321.0 (37.4) |  |  |
| 3 | 58.9 ( 7.0) | 53.0 ( 6.1) |  |  | 75.5 ( 8.8) | 71.0 ( 8.3) |  |  |
| 4 | 9.4 ( 1.1) | 8.0 ( 0.9) |  |  | 6.3 ( 0.7) | 6.0 ( 0.7) |  |  |
| Differentiation (%) |  |  | 0.14 | 0.096 |  |  | 0.989 | 0.007 |
| I | 0.0 ( 0.0) | 4.0 ( 0.5) |  |  | 2.9 ( 0.3) | 3.0 ( 0.3) |  |  |
| II | 290.4 (34.3) | 294.0 (33.9) |  |  | 260.0 (30.2) | 262.0 (30.5) |  |  |
| III | 556.1 (65.7) | 570.0 (65.7) |  |  | 598.0 (69.5) | 594.0 (69.2) |  |  |
| Stage (%) |  |  | 0.945 | 0.107 |  |  | 1 | 0.014 |
| Ia | 93.9 (11.1) | 78.0 ( 9.0) |  |  | 88.5 (10.3) | 90.0 (10.5) |  |  |
| Ib | 202.5 (23.9) | 195.0 (22.5) |  |  | 218.5 (25.4) | 215.0 (25.0) |  |  |
| IIa | 60.2 ( 7.1) | 66.0 ( 7.6) |  |  | 66.6 ( 7.7) | 66.0 ( 7.7) |  |  |
| IIb | 184.9 (21.8) | 221.0 (25.5) |  |  | 231.2 (26.9) | 231.0 (26.9) |  |  |
| III | 211.1 (24.9) | 215.0 (24.8) |  |  | 154.3 (17.9) | 157.0 (18.3) |  |  |
| IV | 93.8 (11.1) | 93.0 (10.7) |  |  | 101.7 (11.8) | 100.0 (11.6) |  |  |
| Biliary drainage (%) | 143.2 (16.9) | 134.0 (15.4) | 0.661 | 0.04 | 148.7 (17.3) | 145.0 (16.9) | 0.836 | 0.01 |
| TB | 15.98 (11.10-65.50) | 16.60 (11.20-64.20) | 0.83 | 0.005 | 16.80 (11.40- 68.27) | 16.50 (11.80- 70.27) | 0.903 | 0.004 |
| AIB | 38.00 (36.00-42.00) | 39.00 (36.00-42.00) | 0.197 | 0.107 | 39.00 (37.00- 43.00) | 40.00 (36.00- 43.00) | 0.763 | 0.003 |
| FBG | 5.93 (4.97-7.36) | 5.97 (5.30-7.32) | 0.464 | 0.014 | 6.04 (5.33- 7.41) | 6.12 (5.43- 7.56) | 0.225 | 0.021 |
| chemotherapy (%) | 455.3 (53.8) | 493.0 (56.8) | 0.54 | 0.06 | 525.0 (61.0) | 524.0 (61.0) | 0.994 | <0.001 |
| CA199 | 217.48 (32.13-840.49) | 151.30 (40.10-484.10) | 0.186 | 0.012 | 150.26 (40.14- 449.00) | 162.50 (40.98- 544.47) | 0.342 | 0.008 |
| ALB, albumin; FBG, fasten blood glucose; TB, total bilirubin; SMD, standard deviation mean difference. | | | | | | | | |
